# Psychological Examination of Political Philosophies: Interrelationship Among Citizenship, Justice, and Well-Being in Japan

**DOI:** 10.3389/fpsyg.2021.790671

**Published:** 2022-02-28

**Authors:** Masaya Kobayashi

**Affiliations:** Graduate School of Social Sciences, Chiba University, Chiba, Japan

**Keywords:** political philosophy, positive psychology, citizenship, justice, well-being, Japan, communitarianism, eudaimonia

## Abstract

This paper examines assumptions concerning the relationship between citizenship, justice, and well-being, based on representative political philosophies, including egoism, utilitarianism, libertarianism, liberalism, and communitarianism. A previous paper raised the possibility of an inter-disciplinary framework for collaboration between psychology and political philosophy. This study picks up that thread and attempts to actualize a collaborative research effort based on a framework grounded in positive political psychology. The first part of this study reflects on the methodology situated between empirical psychology and philosophy in reference to the debates caused by psychological and philosophical situationism. In response to its criticism against virtue ethics, the possibility of reconstructing it on empirical psychology has paradoxically emerged. Similarly, this study validates assumptions on political philosophies employing the psychological method concerning well-being. Accordingly, the central part examines the plausibility of the assumptions by empirical evidence obtained from two internet surveys (2020, *N* = 5000; 2021, *N* = 6885) in Japan. The relationships between citizenship, justice, and well-being are the most substantial in the communitarian assumption. The exploratory factor analysis of the two surveys illuminates that the correlations between citizenship, justice, and well-being (or political well-being) are substantial. This relationship denies the egoism assumption. Moreover, almost all correlations between the three are higher based on virtue-related indicators than hedonic ones. These findings are not in tune with the utilitarian assumption and are most congruent to the communitarian assumption. In addition, citizenship and justice correlate more with political well-being than overall well-being. As these are more directly associated with political well-being in the communitarian assumption, this result aligns with the assumption. Furthermore, the positive relationship between disparity elimination and well-being fits the liberal rather than the libertarian assumption. Nevertheless, the substantial correlation between ethical justice and well-being is higher by virtue-related indicators than hedonic indicators, suggesting distributive justice is associated with the ethical dimension. Again, this fits the communitarian assumption rather than the liberal assumption. Thus, philosophical psychology empirically verifies the interdependence of the three conceptions and the relative plausibility of the communitarian assumption. Moreover, as the relationship between the three is essential for political philosophies, the result increases the reliability of communitarianism.

## Collaborative Research Between Political Philosophy and Psychology

### Interdisciplinary Framework and Well-Being as the Common Conception

This article attempts to conduct collaborative research based on an interdisciplinary framework. The previous one, “Political Philosophies and Positive Political Psychology: Inter-disciplinary Framework for the Common Good,” argues as follows: positive psychology can be associated with not only utilitarianism but also other political philosophies. Moreover, there is correspondence between representative political philosophies and psychology. For example, egoism, libertarianism, and liberalism correspond to psychology as usual; in contrast, liberal perfectionism, utilitarianism, and communitarianism correspond to positive psychology.

Although the concept of well-being has been explored especially in positive psychology, it can be effective for discussing the relationship between these philosophies and psychologies, because well-being is related to objects of almost all philosophies in their ways, whether its conception is hedonic or eudaimonic.

Although egoism, libertarianism, and liberalism principally correspond to “psychology as usual,” they are somehow related to well-being. As egoism pursues a person’s own happiness or self-interest, it is a kind of hedonic well-being. The focus of deontological theories of libertarianism and liberalism is prevention of human rights from their infringement by an authoritarian government, and this is the critical research object of psychology as usual. Nevertheless, these philosophies’ positive aim is to enable people to pursue their own happiness by utilizing their human rights. Therefore, these concern individual well-being.

In contrast, hedonic well-being is the essential component of utilitarian tradition, and communitarianism requires people to hold virtues and, therefore, is closely related to the eudaimonic conception of well-being.

Consequently, some kind of well-being is associated with all the political philosophies mentioned above, and it can work as the common conception of these philosophies. This notion can, thus, work as a common way of measurement in empirical analyses associated with these philosophies. The basic characteristics of the main political philosophies are summarized in Supplementary Appendix 1 regarding the relationship among citizenship, justice, and well-being.

### Empirical Approach of Philosophical Psychology Beyond Methodological Impasse

The last article indicated a prospect of philosophical psychology or psychological philosophy because of interdisciplinary collaboration between the two disciplines. For this purpose, the article outlined major political philosophies and other recent approaches, such as liberal perfectionism, capability approach, and deliberative democracy, and illustrated the configuration of political philosophies and correspondence between these and psychological approaches.

This article intends to explore philosophical (and empirical) psychology in the sense of an empirical psychological investigation inspired or led by philosophical ideas, utilizing the interdisciplinary framework and concept of well-being.

Nevertheless, there is a methodological issue to be discussed beforehand, because there is a rigid difference concerning methodology and epistemology between normative philosophy and descriptive science in the standard view of modern positivistic philosophy and sciences.

This issue has come to be salient after the appearance of the encounter between philosophy and empirical psychology. Typically, there has been a “person-situation debate” caused by psychological situationism in the 1960s and 1970s, and the following “virtue ethics and situationism” debate caused by philosophical situationism in the late 1990s.

Traditional Aristotelian virtue ethics embrace both descriptive observations of human virtues and normative ethics for *eudaimonia*, that is, flourishing. In contrast, psychological situationism, pioneered by Mischel (1968), denied the existence of consistent personal traits because situations influenced human actions: arguments depended on results of well-known experiments (Isen and Levin, 1972; Milgram, 1974) in social psychology at the time. Thus, situationism contradicts classical virtue ethics.

Nevertheless, Mischel himself came to call these debates pseudo-controversies and “heated but futile battles” (Mischel, 2004, p. 4), and the situationist movement in psychology withered. Instead, philosophical situationism surged and attacked virtue ethics (Dorris, 1998; Harman, 1999). Then, there was a significant methodological debate between philosophical situationists and defenders of virtue ethics (Miller, 2003, 2021; Kametekar, 2004; Screenivan, 2008; Croom, 2014).

Through this debate emerged a theoretical possibility of bridging scientific psychological approaches and virtue ethics. For example, the initiator of psychological situationism, Mischel, turned to create a “cognitive-affective personality model” (Mischel and Shoda, 1995). With regard to this, an eminent virtue theorist argued that this model could paradoxically lead to the construction of an empirical approach to personality, friendly to Aristotelian virtue theory, based on social psychology evidence (Russel, 2014). Moreover, there was an attempt to ground a virtue theory in psychological research on social intelligence (Snow, 2010). Following this, it was argued that virtue ethics could be a more empirically adequate moral psychology than alternatives such as utilitarianism and Kantianism (Ciurria, 2014).

These arguments may be a precedent of philosophical psychology or psychological philosophy: this is the product of the unexpected collaboration or dialectical integration between these two fields due to tensioned exchange between criticism and counterargument. It is in this context that the encounter between positive psychology and political philosophy can generate a new attempt at philosophical psychology.

First, positive psychology presents a new psychological descriptive model of character traits: the VIA model’s classification of virtues and character strengths is the scientific renovation of classical virtue ethics. Although there is little philosophical scrutiny other than an exceptional work (Kristjánsson, 2013), recent developments have approached Aristotelian ethics more and more: for example, the concept of optimal use of character strengths in contrast to its overuse and underutilization is a scientific revival of the Aristotelian golden mean (Niemiec, 2019).

Second, there is empirical evidence that virtue or some dispositional character strengths have correlations with well-being (Kesebir and Diener, 2014; Kaufman, 2015; Höfer et al., 2020). This supposition reminds us of the Aristotelian normative argument, whether the science of positive psychology applies the discovery as prescriptive or facilitative.

Thus, positive psychology embraces both empirical theses of dispositions and correlational studies between disposition and well-being: these approximately correspond, respectively, to descriptive and normative Aristotelian virtue ethics.

It goes without saying that there can be various associations between empirical psychology and virtue ethics of various religions or morality in other cultural traditions in the world. Naturally, then, it is desirable to explore them empirically. As the VIA model’s classification in positive psychology has a relatively universal character, it may be possible to conduct such scientific examinations.

Now that empirical investigation of virtue ethics has become possible by the emergence of VIA, an empirical investigation concerning political philosophy would be worthwhile based on positive psychology. Then, this study pursues the psychological approach of political philosophy. In other words, this study attempts philosophical psychology, namely, empirical psychological inquiry inspired by political philosophy.

### Psychological Examination of the Plausibility of Political Philosophies Concerning Their Assumptions

For empirical investigation, it was crucial that, in response to Mischel’s problem presentation, Ed-Diener and others empirically scrutinized the temporal stability and cross-situational consistency of affective, behavioral, and cognitive responses measured by reports of feelings and situations (Diener and Larsen, 1984), and that he established the measurement method of well-being. As there were widespread doubts concerning the credibility of measurement, this validation made the development of happiness studies and positive psychology possible.

Accordingly, it would be possible to empirically examine which assumption deriving from political philosophy matches reality well by introducing well-being. Then, this study examines the relationship between citizenship, justice, and well-being. For example, as Supplementary Appendix 1 indicates, communitarianism supposes a more substantial relationship between citizenship/justice and well-being than egoism, libertarianism, and liberalism. The degree can be estimated empirically. Therefore, it is possible to examine assumptions deriving from political philosophies by the psychological method.

This analysis increases or decreases the relative plausibility of political philosophies, because at least an assumption grounded on political philosophy is demonstrated to fit the reality better or worse in comparison with other assumptions based on other philosophies.

Such an analysis cannot vindicate some political philosophy as a whole, because there can be various assumptions arising from political philosophies. If one assumption grounded on a specific political philosophy best fits the reality, another assumption may not correspond to the actual world. As a result, the specific philosophy may not be adequately attested, because there can be inconsistency regarding the results across various assumptions. Nonetheless, if the assumption analyzed is crucial for political philosophy in general, the result can affect the reliability of the particular political philosophy.

Alternatively, a political philosophy may be most credible about some assumptions, while another may be most presumable regarding another supposition. This inconsistency may lead to a combination of political philosophies relative to issues concerning assumptions. This philosophical reconstruction would be the creation of psychological philosophy, a revised philosophy informed by psychological inquiry.

The methodological issue here is how to measure variables in assumptions. There is a method of objective measurement and subjective measurement. Positive psychology proved that while objective well-being indicators such as GDP are popular in social sciences, subjective well-being indicators are valid and reliable for psychological investigations.

Regarding the issue of this article, the method of measuring well-being, citizenship, and justice is indispensable. The objective way of measuring citizenship is concerned with, for example, the existence of legal citizenship and frequency and degree of exercising active citizenship. Similarly, an objective way of measuring social justice is an indicator of disparity, such as the Gini coefficient.

However, whether subjective methods can measure citizenship and justice has yet to be explored at length. Accordingly, this study attempts to measure the subjective understanding of people concerning the existence or the degree by several simple questions. This method is the most feasible at the moment. The measured conceptions are, as it were, “subjective citizenship” and “subjective justice” like subjective well-being.

It reflects the present stage of political psychology that few preceding pieces of research have scrutinized the consistency and stability of subjective citizenship and subjective justice, in contrast to well-being measurement. Nevertheless, it is worthwhile to conduct empirical analyses by means of subjective measurement as an initial stage of positive political psychology. The following sections elucidate an attempt at philosophical psychology, an empirical psychological inquiry inspired by political philosophies.

## Empirical Study on Positive Political Psychology in Japan

### Two Surveys and Assumptions

#### Purpose

The principal purpose is the correlational analysis of relationships among citizenship, justice, and well-being. Moreover, the ethical dimension important in communitarianism was examined using virtue-related indicators to analyze the correlation between well-being and citizenship/justice.

Two surveys were conducted in Japan in 2020 and 2021 to investigate the relationship among citizenship, justice, and well-being, and examine which assumption led by political philosophy was most plausible.

Several assumptions and hypothetical models from major political philosophies discussed in the previous paper were under verification for this purpose. These philosophies are egoism, utilitarianism, libertarianism, and liberalism: Supplementary Appendix 1 (Table 1 in the previous article) classified the fundamental character. However, as conservatism within the table is not necessarily an academic political philosophy, the following arguments did not address it.

#### Assumption and Method in the Correlational Analysis

In the last section, “Multidisciplinary Development for Common Good as Collective Well-being,” the previous article argued that overall or general well-being embraces both individual and collective aspects, and that, therefore, it was influenced by both individual and collective well-being. Individual well-being is affected by set points based on biological genes, circumstances, and intentional activities of individuals; collective well-being is affected by culture, society (and economy and community), and politics (or policy). Citizenship and justice are concerned with not only individual well-being but also collective well-being, especially political well-being.

Thus, this article principally examined the relationship among citizenship, justice, and well-being utilizing general or overall well-being indicators and political well-being indicators to detect the relationship between citizenship/justice and individual or collective well-being. While political well-being, a constituent of collective well-being, is supposed to be associated with citizenship and justice directly, general or overall well-being is indirectly associated with them through collective well-being.

The discussions summarized in Supplementary Appendix 1 derived from the previous article lead to the following assumptions concerning the relationship between citizenship/justice and well-being for the correlational analysis.^1^

1. Egoism assumption: relationships between citizenship/justice and well-being are non-existent or weak.
2. Utilitarian assumption: hedonic well-being constitutes justice, and the relationship between citizenship and justice (or well-being) is weak or mild.
3. Libertarian assumption: citizenship and justice enable well-being, but the degree depends on a case-by-case assessment. Private citizenship should be firm, but public citizenship may be weak. Justice means legal liberal justice, and it includes civil rights and property rights. Welfare for the poor is unjust if it violates property rights, and it frequently decreases the well-being of people.
4. Liberal assumption: citizenship and justice enable well-being, but the degree depends on a case-by-case assessment. Private citizenship should be firm, and public citizenship is substantial. Nevertheless, citizenship and justice need not be substantial and ethical. Justice contains legal liberal justice and distributive justice. Therefore, welfare for the poor is just to some extent and increases the overall well-being of people.
5. Communitarian assumption: eudaimonic well-being is as essential as hedonic well-being. The relationship between citizenship/justice and comprehensive well-being is substantial. Both private and public citizenship for welfare should be substantial, and citizenship and justice contains an ethical or virtuous character. Justice contains liberal justice, distributive justice, and ethical justice.

Consequentially, the degree of the relationship between citizenship/justice and well-being is assumed to be most substantial in the communitarian assumption and lowest in the egoism assumption. In contrast, the relationship is somewhere apparent between the two in the other three assumptions.

Therefore, the correlations based on empirical evidence in the two surveys will demonstrate which of the presented assumptions will gain more support from perceptions of the public captured in the surveys. In addition, the abovementioned relationship has a connection with the essential tenets of main political philosophies. Therefore, the result of the correlational analysis will predict the relative plausibility of these philosophies to some extent.

Moreover, the relationship between citizenship/justice and well-being would be more robust in using virtue-related indicators than hedonic indicators according to the communitarian assumption, while there is no such supposition in the other assumptions.

Furthermore, comparing the libertarian/liberal assumption with the communitarian assumption, indicators of collective well-being can play a significant role. The degree of the relationship between citizenship/justice and well-being depends on a case-by-case assessment in the former assumptions because the consequences of people’s pursuit of their well-being are influenced by their own situations, abilities, efforts, and luck. Accordingly, these assumptions do not mainly take the effect of the common good into account. In contrast, the effect is an essential element for the communitarian assumption, as well as the effect of individual factors. The subjective level of realizing the common good can be regarded as being associated with collective well-being. Then, citizenship and justice are concerned with collected well-being; accordingly, they may be consequentially associated with the general well-being of individuals in this assumption. The causality concerning the common good explains why the relationship between citizenship/justice and well-being is most substantial in this assumption. Therefore, while the correlation between citizenship/justice and collective well-being is not conspicuous in the libertarian/liberal assumptions, it is vital and plays a decisive role (in comparison with the correlation regarding individual well-being) in the communitarian assumption. This article focuses on political well-being within collective well-being for empirical analysis, because it is most associated with citizenship and justice.

Accordingly, it would be possible to verify whether the communitarian assumption is the most credible by examining the following matters: (1) the correlations between citizenship/justice (or representative items concerned with these) and well-being, including political well-being; (2) the comparison of the correlations concerning well-being by utilizing virtue-related indicators with those by utilizing hedonic indicators.

To this end, the two Internet surveys collected the data through an Internet survey company. The dates and place of these surveys were May 2020 and March 2021 and Japan.^2^ While the first survey (*N* = 5000) was composed of people with no relation to their residential areas within the whole of Japan, those of the second survey (*N* = 6885) consisted of participants that included more than 100 persons within each prefecture (a total of 47 prefectures) in Japan. The format of replies in most of the questions was the 10-grade evaluation. The format associated with the various questionnaires was, thus, coordinated, because it was necessary to integrate the format of the replies because of practical considerations related to the surveys.

#### Instruments

Although there are methodological discussions concerning measurement of well-being (Lee et al., 2021), this study selected several questioners among well-known ones in the present positive psychology, so that a complex, multidimensional, and contextual character of well-being (Ryff et al., 2021) can be measured.

The questions concerning well-being were those in the SWLS (Satisfaction with Life Scale: 5 items), PERMA-profiler (23 items), and I COPPE (16 items). The SWLS, developed by Ed-Diener, is the most common measure in happiness studies and positive psychology (Diener et al., 1985; Diener, 2000). The PERMA-profiler was developed by Butler and Kern (2016) based on the well-known well-being model of Seligman (2011). Prilleltensky et al. (2015) developed the I COPPE for measuring multidimensional well-being in various life domains: overall, psychological, physical, interpersonal, economic, organizational, and community well-being. Our surveys reduce the I COPPE into present and future (5 years later) questions by omitting past because of the practical limit of the number of questions. Instead, these tried to measure political well-being based on the question about “the surrounding political situation” in both surveys.^3^ Furthermore, in addition to covering the overall well-being in seven items of the original I COPPE, this study calculated the averages of the original seven and eight items (including political well-being) as an approximate measure of comprehensive multidimensional well-being in various spheres. Accordingly, this article will abridge overall well-being and political well-being as well as an average of seven items and eight items as I COPPE o/p/7/8 hereafter.

The questions also include those in the HEMA-RX (Hedonic and Eudaimonic Motives for Activities: 16 items) and the original questions of virtues for measuring eudaimonic elements. Veronika Huta developed the HEMA-RX for measuring hedonic and eudaimonic orientation (Huta, 2016). The former orientation is abridged as EUD, while the latter is HED in this article. The original six questions simply ask participants to have six virtues (intellect, courage, humanity, justice/fairness, temperance, and transcendence) enumerated in the VIA (Values in Action Inventory of Strength; Peterson and Seligman, 2004). This study regards the sum of numbers in their answers corresponding approximately to the subjective quantity of their total virtues. This indicator of “comprehensive virtues” will be called CV hereafter.

Moreover, as the questions include some items simply asking about satisfaction or happiness, the average of each related item is expressed, respectively, as SAT and HAP.^4^ EUD and CV measure the level of ethical or moral virtuousness: these are the virtue-related indicators, measuring eudaimonic well-being and comprehensive virtue respectively. In contrast, SAT and HAP are the hedonic indicators, which measure simple affective satisfaction and happiness respectively. The SWLS and PERMA are more or less hybrid indicators concerning the well-being indicators, because they include some eudaimonic or non-hedonic elements in, for example, the cognitive or evaluative element in the SWLS and M (meaning) in PERMA.^5^

Public matters in social and political spheres are selected and contrived by examining several indexes and surveys concerning social or public well-being (such as the OECD well-being framework and Human Development Index of UN, Gross National Happiness of Bhutan). The questions included rewarding emotion, income, leisure, education, culture, security, community, trust (social capital), natural environment, diversity, and digitalization.

Among these questions the following 15 items are related to citizenship and justice in both surveys. These are questions about the participants’ subjective recognition of the existence and degree of the following matters. Table 1 indicates the contents of the following question.

**TABLE 1:** Original questions in the two surveys.

		Survey1	Survey2

**Citizenship**			
1. Efficacy	Civil efficacy	How much do you think you can change the society and politics around you in a desirable direction through your involvement?	Do you want to change the society and politics around you in a desirable direction through your involvement?
	Electoral efficacy	Do you think you can change society and politics through elections and referendums in the society around you?	Do you think you can change society and politics through elections and referendums in the society around you?
2. Liberty and rights	Political liberty (freedom enabling articulation of opinions)	I think I have the political liberty to express my opinion.	Do you think there is political liberty in Japan to express one’s opinion?
	(Respect of) human rights	I believe that fundamental human rights are respected in my country.	Do you think that fundamental human rights are respected in Japan?
3. Trust and rulemaking	Political trust (trust in politicians in communities)	Do you think that you can trust politics and politicians in the society around you?	Do you think that you can trust the politics and politicians in your community?
	Administrative trust (trust in administration in communities)	Do you think that you can trust the administration (government and local government) in the society around you?	Do you think that you can trust the Japanese administration (government and local government)?
	Rulemaking (functioning of citizen’s rulemaking)	Do you think that citizen-led activities to formulate and change rules (e.g., referendums, signatures, online and offline expression of will, etc.) are functioning in the society around you?	Do you think that citizen-led activities to formulate and change rules (referendums, signatures, lobbying political parties and politicians, etc.) are functioning in the society around you?

**Justice**			

4. Disparity	(Recognition of) disparity	How much disparity do you think exists in the society around you?	Do you think that there is disparity in the society around you?
	Economic disparity	Do you think there are economic disparities in the society around you based on your income and assets?	Do you think that there are economic disparities in the society around you based on your income and assets?
	Disparity chain (intergenerational chain of disparity)	Do you think there is a “chain of disparity” in the society around you, such that economic disparity leads to further disparity in education and occupation?	Do you think there is a “chain of disparity” in the society around you, such that disparity leads to further disparity in education and occupation?
	Opportunity disparity (disparity of opportunity)	Do you think that disparities in learning opportunities and employment opportunities occur in the society around you due to your family environment, race, or assets inherited from your parents’ generation, which you cannot change through your efforts?	Do you think that disparities in learning opportunities and employment opportunities occur in the society around you due to your family environment, race, or assets inherited from your parents’ generation, which you cannot change through your efforts?
	Disparity elimination (elimination of disparity)	Do you think that the society around you realizes the elimination of disparities (equal society) through social welfare and redistribution through taxes?	Do you think that the society around you realizes the elimination of disparity (equal society) through social welfare and redistribution through taxes?
5. Ethical justice	Non-corruptive fairness	I think that my government is corruption-free and fair.	Do you think that the Japanese government is corruption-free and fair?
	Justness (justice and fairness)	I believe that fairness and justice are achieved in our country’s politics in terms of decision-making and disparity between rich and poor.	Do you think that Japanese politics achieves fairness and justice in terms of decision-making and the disparity between rich and poor?
	Virtuous politicians (virtuous characters of national politicians)	I believe that politicians in my country are generally of good character.	Do you think that Japanese politicians are generally of good character?

**Citizenship** (7:2 + 2 + 3):

1. Efficacy (2): civil efficacy and electoral efficacy (possibility of change by electoral participation).
2. Liberty and rights (2): political liberty (freedom enabling articulation of opinions) and (respect of) human rights.
3. Trust and rulemaking (3): political trust (trust in politicians in communities), administrative trust (trust in administration in communities), and rulemaking (functioning of citizen’s rulemaking).

**Justice** (8:5 + 3):

4. Disparity (5): (recognition of) disparity, economic disparity, disparity chain (intergenerational chain of disparity), opportunity disparity (disparity of opportunity), and disparity elimination (elimination of disparity).
5. Ethical justice (3): non-corruptive fairness, justness (justice and fairness), and virtuous politicians (virtuous characters of national politicians).

Subcategories of citizenship and justice are only for convenience. Later analyses selected relevant items from these. Efficacy (1) and Liberty/rights (2) will function as items of political citizenship and legal citizenship/justice, respectively; Disparity (4) and Ethical justice (5) will function as items of distributive justice and ethical justice, respectively.

Finally, the following study utilized SPSS (ver.27) of IBM for statistical calculations.

#### Participants

As a result of the residential difference mentioned in section “Assumption and Method in the Correlational Analysis,” the ratio of participants in several prefectures, including big cities, is much higher in the first survey than in the second survey: 75.6% (first survey): 35.4% (second survey). In contrast, the number of males/females, respective age cohort, married/unmarried (including separation by divorce or death) is coordinated equally only in the first survey.

Table 2 summarizes basic features of the questionnaires and respondents. The questions of the two surveys focus on psychological, political, economic, and social matters. For example, psychological questions are related to well-being, while political, economic, and social items are sometimes concerned with citizenship and justice.

**TABLE 2:** Participants of the two studies.

	Survey1 (%)	Survey2 (%)
*N*	5000	6885
Number of questions	383	401
**Residence**
16 prefectures with big cities	3780 (75.6)	2435 (35.4)
32 prefectures without big cities	1220 (24.4)	4450 (64.6)
**Sex**		
Male	2500 (50)	4427 (64.3)
Female	2500 (50)	2458 (35.7)
**Age**		
10’s	834 (16.6)	37 (0.5)
20’s	834 (16.6)	460 (6.7)
30’s	833 (16.6)	1043 (15.1)
40’s	833 (16.6)	1738 (25.2)
50’s	833 (16.6)	1750 (25.4)
60’s	833 (16.6)	1238 (18.0)
70’s and more		619 (9.0)
**Marital status**		
Married	2294 (45.9)	4091 (59.4)
Unmarried	2469 (49.4)	2254 (32.7)
Separation	237 (4.7)	540 (7.9)*
**Occupation**		
Executive of company or association	46 (0.9)	124 (1.8)
Office worker, staff of association	1513 (30.3)	2097 (30.5)
Part-time employee, contract employee, dispatched labor	248 (5.0)	410 (6.0)
Part-time worker, part-time job, home-based workers without an employment contract	586 (11.7)	806 (11.7)
Civil servants	153 (3.1)	257 (3.7)
Self-employed, family employee, free lance	302 (6.0)	822 (11.9)
Faculty member		123 (1.8)
Student	837 (16.7)	96 (1.4)
Homemaker	718 (14.4)	767 (11.1)
Pensioner	151 (3.0)	603 (8.8)
None	393 (7.9)	693 (10.1)
Others	53 (1.1)	87 (1.3)
**Education**		
Currently attending high school	373 (7.5)	43 (0.6)
Currently attending vocational college, specialized training college	80 (1.6)	84 (1.2)
Currently attending junior college, college	49 (1.0)	47 (0.7)
University/college preparatory school	15 (0.3)	4 (0.1)
Currently attending university	381 (7.6)	89 (1.3)
Currently attending Master’s or Doctoral course	25 (0.5)	19 (0.3)
Junior high school	73 (1.5)	175 (2.5)
High school	1069 (21.4)	2164 (31.4)
Vocational college, specialized training college	389 (7.8)	644 (9.4)
Junior college, college	418 (8.4)	598 (8.7)
University	1912 (38.2)	2669 (38.8)
More than Master’s degree	216 (4.3)	349 (5.1)

*Divorce 419 (6.1)/death 121 (1.8).

#### Procedures

The first survey was a general survey of well-being and social/political situations before the launch of the research topic “Psychology for the Common Good: The Interdependence of Citizenship, Justice, and Well-being across the Globe.” As mentioned above, the survey contains 15 relevant items, which are included in the second survey for well-being and justice/fairness. There are subtle modifications of the expression in questions for attuning it with the other items.

After writing the first draft of the previous article, various statistical analyses were conducted to examine the assumptions above. First, an exploratory factor analysis of items in the two studies searched for factors regarding citizenship and justice.

Second, the following analysis examined the correlations among the extracted factors and well-being, including political well-being, utilizing the indicators of SWLS, the general well-being in PERMA-profiler, and I COPPE (o/p/7/8).

Third, at the same time, the analysis measured the correlations between the extracted factors and well-being, including political well-being, by virtue-related indicators of EUD, and CV and hedonic indicators of HED, SAT, HAP. In contrast, this study regards SWLS and PERMA as hybrid well-being indicators, measuring both the hedonic and anhedonic elements.

Finally, the significant correlations mentioned above in both surveys were checked by partial correlation analysis, removing the effect of controlling ascriptive variables such as sex, age, marriage, education, residence, employment, and income.

## Exploratory Factor Analysis and Correlations in Survey1

### Analysis 1-1: Two Factors of Citizenship/Justice and Disparity

The common factor analysis of items of citizenship and justice (maximum likelihood method, Promax rotation, eigenvalue greater than 1) extracted two factors in the first survey (Table 3). These represent “1. Citizenship and justice, 2. Disparity.” Accordingly, there is some commonality between citizenship and justice. Inter-factor correlation is −0.144, suggesting that the recognition of disparity decreases citizenship/justice.

**TABLE 3:** Two factors and correlations in study 1.

	Factor	Citizenship and justice	Disparity
Exploratory factor analysis (maximum likelihood method, Promax rotation, eigenvalue greater than 1)	Items (factor loading > 0.3)	civil efficacy, electoral efficacy, political trust, administrative trust, rulemaking, disparity elimination, political liberty, human rights, non-corruptive fairness, justness, virtuous politicians	disparity, economic disparity, disparity chain, opportunity disparity
Factor correlation matrix	Citizenship and justice	1	−0.144
	Disparity	−0.144	1
Correlation between factor scores*	Citizenship and justice	1	−0.157
	Disparity	−0.157	1
Correlation with well-being	SWLS	0.545	−0.008
	PERMA (general WB)	0.503	0.129
	I COPPE (o/p)	0.473/0.650	0.090/−0.028
	I COPPE (7/8)	0.530/0.561	0.088/0.075
	SAT	0.476	0.054
	HAP	0.432	0.085
Correlation with virtue	CV	0.531	0.195
Correlation with orientation	EUD	0.374	0.268
	HED	0.130	0.330

*N = 5000.*

*This line and below indicate the correlations between items in the second column and factor scores (of the factors in the first line). In most cases, p < 0.001; only **p(0.046) < 0.05.

The following correlations are all significant (*p* < 0.001, with one exception, indicated later). As Table 3 indicates, the first factor (citizenship and justice) and well-being are moderate (SWLS: 0.545; PERMA: 0.503; I COPPE o/p/7/8: 0.473/0.65/0.53/0.561). The correlation between the first factor and CV is also moderate (0.531). It is higher than the case of SAT (0.476) and HAP (0.432), just as the correlations regarding SWLS and PERMA are higher than SAT and HAP. The correlation between it and EUD (0.374) is higher than HED (0.130).

On the other hand, the correlation between disparity and well-being is much less than the correlation above, negative or low [SWLS −0.008; PERMA: 0.129, I COPPE o/p/7/8: 0.09/−0.028 (*p* < 0.05)/0.088/0.075]. The correlation between the second factor and CV is positive but low (0.195) and higher than the cases of SAT (0.054) and HAP (0.085). The correlation between it and EUD (0.268) is lower than that of the corresponding HED (0.33).

It follows from these that citizenship and justice are moderately related to well-being, and that the degree of the relation is higher according to the virtue-related indicators (EUD and CV) than the hedonic indicators (SAT and HAP). On the other hand, there is a negative relationship between the disparity and citizenship/justice. It is still positively related to well-being (except SWLS), but the degree is low. Although it is positive but low concerning the virtues, the hedonic orientation is more associated than the eudaimonic orientation.

In addition, the correlation between I COPPE-o and I COPPE-p was relatively high (0.677), as has been expected. The correlation (0.65) between citizenship/justice and political well-being (I COOPE-p) is higher than that (0.473) concerning overall well-being (I COPPE-o). As a result, the correlation (0.561) regarding I COPPE8 is higher than that (0.530) of I COPPE7. As citizenship and justice are more related to political well-being than overall well-being, these results are understandable. On the other hand, the correlation between disparity and political well-being is negative (−0.028), while the correlation concerning overall well-being is slightly positive (0.09). This finding may suggest that people tend to feel disparity as politically undesirable than generally undesirable.

### Analysis 1-2: Three Factors of Citizenship and Justice

The factor plot indicated that it was possible to distinguish items concerning justice from those associated with citizenship. Accordingly, fixing the three factors extracted two factors corresponding to citizenship and justice, and the disparity factor was also associated with justice.

Table 4 indicates the following result: according to the inter-factor correlations, there is a positive relationship between citizenship and justice; there is a negative correlation between the two and disparity. It is reasonable that while there is a positive correlation between citizenship and justice, these are negatively associated with disparity. The correlation of the former two (citizenship and justice) is impressively high (0.716).

**TABLE 4:** Three factors and correlations in study 1.

	Factor	Citizenship	Disparity	Justice
Exploratory factor analysis (maximum likelihood method, Promax rotation, designation of three factors)	Items (factor loading > 0.3)	civil efficacy, electoral efficacy, political trust, administrative trust, rulemaking, disparity elimination, political liberty	disparity, economic disparity, disparity chain, opportunity disparity	non-corruptive fairness, justness, virtuous politicians
Factor correlation matrix	Citizenship	1	−0.063	0.716
	Disparity	−0.063	1	−0.212
	Justice	0.716	−0.212	1
Correlation between factor scores*	Citizenship	1	−0.072	0.791
	Disparity	−0.072	1	−0.236
	Justice	0.791	−0.236	1
Correlation with well-being	SWLS	0.525	0.008	0.496
	PERMA (general WB)	0.503	0.142	0.415
	I COPPE (o/p)	0.469/0.630	0.103/−0.010**	0.400/0.589
	I COPPE (7/8)	0.528/0.556	0.102/0.090	0.446/0.477
	SAT	0.471	0.067	0.406
	HAP	0.430	0.096	0.361
Correlation with virtue	CV	0.536	0.210	0.434
Correlation with orientation	EUD	0.386	0.279	0.286
	HED	0.147	0.335	0.069

*N = 5000.*

*This line and below indicate the correlations between items in the second column and factor scores (of the factors in the first line). In most cases, p < 0.001; only **p(0.471): non-significant.

The correlation between citizenship and well-being is also moderate, while that between justice and well-being is also moderate. The correlation between justice/citizenship and CV is also moderate, and, respectively, higher than SAT and HAP, as the corresponding SWLS and PERMA are higher than SAT and HAP. On the other hand, the correlation between these two factors and EUD is higher than the low correlation regarding HED.

On the other hand, the correlation between disparity and well-being is much less than that above, negative or low, although I COPPE-p is non-significant because of the low degree of p. The correlation between disparity and CV is positive but relatively low, and it is higher than the cases of SAT and HAP. The correlation between disparity and EUD is lower than that between it and HED.

The almost same conclusions followed from these analyses between citizenship, justice, and well-being as the analysis in the last section “Analysis 1-1: Two Factors of Citizenship/Justice and Disparity.” The only slight difference is that while disparity is not positively related to well-being only in SWLS in the last analysis, it is positively related even in SWLS in this analysis.

The finding regarding political well-being is almost the same as well. The correlation between citizenship and political well-being (I COPPE-p) is higher than that concerning overall well-being (I COPPE-o); the correlation regarding citizenship and justice is also higher than overall well-being. As a result, the respective correlations regarding I COPPE8 are higher than those of I COPPE7. Although the correlation between disparity and I COPPE-p is negative but non-significant, the tendency regarding citizenship and justice seen in the last section appears again.

### Analysis 1-3: Six Factors of Citizenships and Justice

As the subsequent analysis designated the numbers of factors above three, there emerged other factors one by one and up to six, sometimes accompanied by the split of some existing factors: first, that of liberty and rights; second, that of civil efficacy; third, that of the disparity elimination (the fourth, sixth, and fifth factors, respectively, among the six) (Table 5). While the factor of liberty/rights corresponds to the liberal justice and legal citizenship mentioned above, the factor of disparity elimination corresponds to distributive justice. Civil efficacy ramified from the original factor of citizenship, and the original factor of justice shrank into ethical justice. Thus, the two factors of citizenship and civil efficacy correspond to the original and broad concept of citizenship; the other four factors of disparity, ethical justice, liberty/rights (liberal justice), and disparity elimination (distributive justice) correspond to justice in general.

**TABLE 5:** Six factors and correlations in study 1.

	Factor	Citizenship	Disparity	Ethical justice	Liberal justice /citizenship (liberty and rights)	Distributive justice	Civil efficacy
Exploratory factor analysis (maximum likelihood method, Promax rotation, designation of six factors)	Items (factor loading > 0.3)	civil efficacy, electoral efficacy, political trust, administrative trust, rulemaking	disparity, economic disparity, disparity chain, opportunity disparity	non-corruptive fairness, justness, virtuous politicians	political liberty, human rights	disparity elimination	civil efficacy
Factor correlation matrix	Citizenship	1	−0.085	0.751	0.514	0.602	0.334
	Disparity	−0.085	1	−0.246	0.175	0.033	0.060
	Ethical justice	0.751	−0.246	1	0.491	0.561	0.240
	Liberal justice	0.514	0.175	0.491	1	0.376	0.393
	Distributive justice	0.602	0.033	0.561	0.376	1	0.175
	Civil efficacy	0.334	0.060	0.240	0.393	0.175	1
Correlation between factor scores*	Citizenship	1	−0.095	0.813	0.596	0.735	0.492
	Disparity	−0.095	1	−0.265	0.196	0.037	0.072
	Ethical justice	0.813	−0.265	1	0.569	0.687	0.364
	Liberal justice	0.596	0.196	0.569	1	0.509	0.558
	Distributive justice	0.735	0.037	0.687	0.509	1	0.346
	Civil efficacy	0.492	0.072	0.364	0.558	0.346	1
Correlation with well-being	SWLS	0.495	0.008**	0.494	0.597	0.435	0.505
	PERMA (general WB)	0.466	0.140	0.410	0.667	0.421	0.553
	I COPPE (o/p)	0.434/0.603	0.101/−0.008***	0.395/0.593	0.617/0.625	0.394/0.512	0.495/0.497
	I COPPE (7/8)	0.491/0.520	0.100/0.088	0.442/0.474	0.676/0.687	0.440/0.462	0.545/0.554
	SAT	0.439	0.064	0.403	0.616	0.390	0.490
	HAP	0.398	0.093	0.355	0.608	0.347	0.471
Correlation with virtue	CV	0.498	0.210	0.429	0.650	0.438	0.558
Correlation with orientation	EUD	0.349	0.277	0.276	0.555	0.313	0.470
	HED	0.119	0.331	0.053	0.402	0.152	0.213

*N = 5000.*

*This line and below indicate the correlations between items in the second column and factor scores (of the factors in the first line). In most cases, p < 0.001; only **p(0.569): non-significant. ***p(0.561): non-significant.

The inter-factor correlation matrix indicates a high correlation between the first factor (citizenship) and the three factors concerning justice (ethical, liberal, and distributive justice); there are relatively high correlations among the three factors of justice. The other two factors (disparity and civil efficacy) are relatively lowly or negatively correlated with the four abovementioned factors.

Table 5 indicates the following results: the correlation between citizenship/civil efficacy and well-being is also moderate, while the correlations regarding ethical/liberal/distributive justice and well-being are moderate. In particular, the correlations between liberal justice (liberty/rights) and well-being are highest.

The correlations between citizenship/civil efficacy or the other three factors of justice and CV are, respectively, moderate and higher than the SAT and HAP in all cases without any exception. Moreover, the correlations concerning CV are higher than those concerning SWLS and PERMA, with only two exceptions (correlation between ethical justice and SWLS, and between liberal justice and PERMA). All of the correlations between these factors and EUD are higher than that between these and HED.

In contrast, the correlations between disparity and well-being are low, although only the SWLS is non-significant. The correlation between disparity and CV is lowest among the six factors and CV, although it is higher than the correlation regarding SAT or HAP. The correlation between it and EUD is lower than that concerning HED.

Nearly the same conclusions followed from these analyses between the six factors and well-being as the analysis in the last section “Analysis 1-2: Three Factors of Citizenship and Justice.” Citizenship/civil efficacy and ethical/liberal/distributive justice are also moderately related to well-being. The degree of the relationship concerning citizenship/civil efficacy and ethical/liberal/distributive justice is higher by the virtue-related indicators than the hedonic indicators. The correlations regarding disparity are exceptional: those regarding well-being are low, and those regarding virtue orientation are lower than hedonic orientation.

The findings of political well-being are also the same as the last section. All correlations between the five items of citizenship and justice (except disparity) and political well-being are higher than those concerning overall well-being. As a result, all correlations regarding I COPPE8 are higher than those of I COPPE7.

### Analysis 1-4: Partial Correlation Analysis Concerning Ascriptions

At the end of analysis 1, partial correlation analysis checked the correlations in analysis 1-3 by removing the effects of controlling variables concerning ascriptions: sex, age, level of education, marriage (married or unmarried), residence (prefecture with or without big cities), and work (employed or unemployed).

As a result, all significant correlations in Table 5 are also significant, and their correlation coefficients are very close to the corresponding partial correlation coefficients. Furthermore, only two insignificant correlations in Table 5 are also only insignificant correlations in this analysis. Thus, this analysis shows that these ascriptive elements did not influence the correlations and conclusions above.

## Explorative Factor Analysis and Correlations in Survey2

### Analysis 2-1: Four Factors of Citizenships and Justice

The result of exploratory factor analysis in the second survey was basically similar to that of the first survey, but a few factors include both citizenship and justice. For example, when the same method (maximum likelihood method, Promax rotation) extracted three factors, the first factor was “justice and citizenship,” while the second and the third were “disparity” and “liberal justice.” The undifferentiation of justice and citizenship is understandable, because these are highly associated even in the first survey. Thus, the undifferentiation is itself a result of the interdependence between citizenship and justice.

Nevertheless, the alternative factor analysis (principal factor analysis, Promax rotation, designating four factors) separated these two: the first factor is justice, the second factor is disparity, the third factor is citizenship, and the fourth factor is civil efficacy. The two factors of justice and disparity are related to justice in a broad sense, and the other two factors of citizenship and civil efficacy correspond to citizenship in general.

As a consequence of calculating the correlations between these factors and well-being in the same way as section “Exploratory Factor Analysis and Correlations in Survey1,” the second analysis results are almost the same (Table 6). This analysis and results are similar to analysis 1-2 of the three factors in the first survey in section “Analysis 1-2: Three Factors of Citizenship and Justice.” The three factors there correspond to the first three factors in this analysis. The fourth factor of civil efficacy was included in citizenship in analysis 1-2 and consisted of only one item of civil efficacy in this analysis. As a result, the following analysis is centered on the first three factors.

**TABLE 6:** Four factors and correlations in study 2.

	Factor	Justice	Disparity	Citizenship	Civil efficacy
Exploratory factor analysis (principal component analysis, Promax rotation, designation of four factors)	Items (factor loading > 0.3)	non-corruptive fairness, justness, virtuous politicians, disparity elimination, political trust, administrative trust, electoral efficacy, rulemaking	disparity, economic disparity, disparity chain, opportunity disparity	electoral efficacy, rulemaking, political liberty, human rights, disparity elimination (−)	civil efficacy
Component correlation matrix (=correlation between factor scores)	Justice	1	−0.118	0.622	0.394
	Disparity	−0.118	1	0.107	0.277
	Citizenship	0.622	0.107	1	0.351
	Civil efficacy	0.394	0.277	0.351	1
Correlation with well-being*	SWLS	0.543	0.046	0.428	0.46
	PERMA (general WB)	0.433	0.256	0.423	0.566
	I COPPE (o/p)	0.414/0.615	0.167/0.084	0.370/0.475	0.491/0.481
	I COPPE (7/8)	0.491/0.521	0.191/0.182	0.440/0.458	0.563/0.568
	SAT	0.475	0.135	0.431	0.500
	HAP	0.339	0.201	0.366	0.463
Correlation with virtue	CV	0.431	0.321	0.430	0.628
Correlation with orientation	EUD	0.379	0.332	0.383	0.601
	HED	0.318	0.355	0.374	0.506

*N = 6885.*

*This line and below indicate the correlations between items in the second column and factor scores (of the factors in the first line). In all cases, p < 0.001.

According to the component correlation matrix, there is a positive relationship among justice, citizenship, and civil efficacy, while there is a negative correlation between the three and disparity. The correlation between justice and citizenship is high (0.622).

Table 6 indicates the following results: first, the correlation between justice and well-being, and that between citizenship and well-being is moderate. Second, the correlation between justice/citizenship and CV is also moderate and higher than HAP,^6^ as SWLS and PERMA correlations are higher than HAP. Finally, the correlations between these two factors and EUD are higher than the correlations regarding HED.

On the other hand, the correlation between disparity and well-being is much less than that above. In contrast, the correlation between disparity and CV is positive, and it is higher than the cases of SAT and HAP. On the other hand, the correlation between it and the EUD is lower than that regarding HED.

Broadly, the same conclusions followed from these analyses between the three factors and well-being as analysis 1 (sections “Analysis 1-1: Two Factors of Citizenship/Justice and Disparity,” “Analysis 1-2: Three Factors of Citizenship and Justice,” and “Analysis 1-3: Six Factors of Citizenships and Justice”). In the same vein, the findings of political well-being are also the same as sections “Analysis 1-1: Two Factors of Citizenship/Justice and Disparity,” “Analysis 1-2: Three Factors of Citizenship and Justice,” and “Analysis 1-3: Six Factors of Citizenships and Justice.” The correlation between justice and political well-being is higher than the correlation concerning overall well-being; the correlation regarding citizenship is also higher than the correlation concerning overall well-being. As a result, the respective correlations regarding I COPPE8 are higher than those of I COPPE7.

Thus, the tendency regarding citizenship and justice seen in section “Exploratory Factor Analysis and Correlations in Survey1” appeared again. Consequently, the analysis in the second survey confirmed the results of analyses 1-1, 1-2, and 1-3.

### Analysis 2-2: Partial Correlation Analysis Concerning Ascriptions

At the end of analysis 2, partial correlation analysis checked the correlations in analysis 2-1 by removing the effects of controlling variables concerning ascriptions: sex, age, level of education, level of income,^7^ marriage (married or unmarried), residence (prefecture with or without big cities), and work (employed or unemployed).

As a result, all significant correlations in Table 6 are significant, and their correlation coefficients are very close to the corresponding partial correlation coefficients. This analysis shows that these ascriptive elements did not influence the correlations and, therefore, conclusions above.

## Discussion: Validity of the Communitarian Assumption

### Existence of Interdependence: Denial of Egoism Assumption

Analyses 1 and 2 unanimously demonstrate substantial relationships between citizenship and justice and between citizenship/justice and well-being, including political well-being. In short, there is substantial interdependence among citizenship, justice, and well-being (or political well-being).

Therefore, the egoism assumption that the relationship between citizenship/justice and well-being is non-existent or weak contradicts the results. In contrast, this does not necessarily deny the assumptions of the other political philosophies, because they admit or argue that there can be more or less of such relationships. Since the communitarian assumption supposes the most substantial interdependence compared with other assumptions, it matches the result to the greatest extent.

### Virtuousness and Well-Being: Denial of the Utilitarian Assumption

The demonstrated substantial interdependence does not fit the utilitarian assumption particularly well because of its supposition that the relationship between citizenship and justice (or well-being) is weak or mild.

Moreover, the results of analyses 1 and 2 unanimously indicate that the correlations between citizenship/justice and well-being are higher according to virtue-related indicators than hedonic ones. This fact verifies that the element of ethical morality is associated with these. Therefore, this result does not support the utilitarian assumption based on hedonic well-being.

On the contrary, this point is in tune with the communitarian assumption, because it emphasizes eudaimonic well-being. The libertarian and liberal assumptions do not particularly match the results, but it does not directly follow that they are wrong, because they admit that individuals respect morality in their private lives and do not necessarily exclude the ethical elements.

### Disparity and Well-Being: Limited Support for the Libertarian Assumption

The demonstrated substantial interdependence does not fit the libertarian and liberal assumption particularly well, because the relationship between citizenship or justice and well-being remains an enabling possibility. Nevertheless, it is difficult to judge the plausibility of libertarian and liberal assumptions by the result, because the assumption allows some degree of interdependence.

Most of analyses 1 and 2 indicate that the correlations between disparity and well-being/virtues are low but positive regarding the distributive issue. The positive correlation suggests that recognizing inequality does not necessarily reduce the well-being of people, in opposition to radical egalitarianism, including communism and socialism. Libertarians reversely suppose that the difference in income may result from fair competition in market economy. They tend to believe that the existence of economic disparity is proof that the market system operates fairly and leads to happiness of people.

In addition, the unanimous result that the correlations between disparity and virtue-related indicators are lower than those concerning the hedonic indicators indicates that the hedonic orientation may enable people to recognize and accept disparity, and that the eudaemonic orientation may hinder recognition. As libertarians tend to pursue their hedonic well-being, they can recognize and accept disparity more easily than non-libertarians. Therefore, this finding fits this libertarian worldview.

### Distributive Justice and Well-Being: Denial of Libertarian Assumption and Support for Liberal Assumption

Nevertheless, the correlation between disparity and well-being does not strongly support the libertarian assumption, because it is low. In addition, there are some cases, including analysis 1-1, where the correlation is sometimes negative. Accordingly, people’s acceptance of disparity does not hinder their happiness, but most do not feel much happier.

On the other hand, there are negative correlations between disparity and citizenship/justice in most cases (analyses 1-1, 1-2, 2-1, and some in 1-3). This result is understandable, because many people think that inequality is an injustice, and that it is not in tune with citizenship.

At the same time, there is a moderately positive relationship between distributive justice (disparity elimination) and well-being in analysis 1-3: people tend to feel happiness in recognizing the decrease in disparity.

Therefore, distributive justice, to some extent, is associated with the well-being of people. This relationship is contradictory to the libertarian assumption and supports the liberal assumption. The latter triumphs over the former by the evidence on this heated fundamental issue of politics, at least in beliefs of the people.

### Ethical Justice and Well-Being: Plausibility of Communitarian Assumption Over Liberal Assumption

Moreover, the correlations are higher on the virtue-related indicators than on the hedonic indicators in analysis 1-3. That is to say, the elimination of excessive disparity is concerned with the increase in well-being, especially in terms of its ethical dimensions.

Accordingly, the ethical element influences the relationship between distributive justice (disparity elimination) and well-being. The fact mentioned above seems to reflect the influence. In short, moralistic or eudaimonic persons tend to feel happier in seeing the decrease in disparity than hedonic persons do.

This result sounds reasonable in the popular understanding of human beings, but it is not remarkably consistent with the liberal philosophy that some ethical or moral worldview should not influence justice. Instead, the communitarian assumption is in tune with this finding.

This point is even more salient in terms of ethical justice. The justice factor in analyses 1-1 and 1-2 transformed into ethical or moral justice in analysis 1-3 in the first survey. Moreover, this ethical factor (Ethical justice) has somewhat high or moderate inter-factor correlations with Citizenship (0.751) and Distributive justice (0.561). It has moderate correlations with well-being, and its correlations measured by the virtue-related indicators are higher than those by hedonic indicators.

That is to say, there is a substantial relationship between the realization of ethical or moral justice and well-being, and the interrelation is clearer using virtue-related indicators. The importance and influence of ethical justice do not suit liberalism well because, in their theory, values should not influence justice, and merely legal rights should decide what justice is in the liberal’s viewpoint.

On the contrary, this finding increases the reliability of the communitarian assumption that ethical justice is related to well-being. Moreover, several of the results mentioned above are consistent with its assumption: substantial interdependence is seen among citizenship, justice, and well-being, and the virtue-related indicators indicate the higher values of interrelations between well-being and citizenship/justice, including distributive justice.

Consequently, analyses 1 (especially 1–3) and 2 verify the plausibility of the communitarian assumption over the other political philosophies, including liberalism.

Moreover, citizenship and justice (except disparity) correlate more with political well-being than with overall well-being. As was mentioned, political well-being is collective well-being most related to citizenship and justice, and collective well-being is associated with the common good in the communitarian assumption. Then, political or collective well-being is directly associated with citizenship and justice in the assumption, and individual well-being is indirectly related to citizenship and justice principally through collective well-being. Therefore, it is reasonable that citizenship and justice (except disparity) correlate more with political well-being than with overall well-being from the perspective of communitarianism, and this result is in tune with the communitarian assumption. In addition, this finding confirms the value of the conception of political well-being.

## Conclusion: Verification by Positive Political Psychology

### Comparative Plausibility of Communitarian Assumption

There are two elements to the interdependence among citizenship, justice, and well-being: whether such interdependence exists and how they are. The examination by positive political psychology enabled us to verify various philosophical arguments scientifically. In addition, the concept of political well-being proved to be helpful in the process of the investigation.

First, the exploratory factor analysis of the two surveys illuminated that the correlations among citizenship, justice, and well-being were substantial. Therefore, the demonstrated result of the interdependence explicitly denies the egoism assumption supposing non- or weak interdependence.

Second, almost all correlations among the three are higher by virtue-related indicators than by hedonic ones. Again, this result does not match the utilitarian assumption.

Third, although the positive correlations between disparity and well-being/virtues match the libertarian assumption, this finding does not validate the assumption firmly because the correlation is low.

Fourth, there is a negative correlation between disparity and citizenship/justice and a moderately positive correlation between disparity elimination and well-being: these findings align not with the libertarian assumption but with the liberal assumption.

Fifth, there is a substantial relationship between the realization of ethical or moral justice and well-being. In addition, citizenship and justice (except disparity) correlate more with political well-being than with overall well-being. These facts are in tune with the communitarian assumption rather than with the liberal assumption. Moreover, almost all the correlations mentioned above are clearer using the virtue-related indicators than using the hedonic ones. Again, this recurrent tendency reinforces the plausibility of the communitarian assumption.

In sum, some results explicitly contradict the assumptions of either egoism or utilitarianism; other findings do not fit either libertarianism or liberalism well. In contrast, all the results do not negate the communitarian assumption, and some support it thoroughly. Therefore, these findings are most congruent to the communitarian assumption than the other philosophical assumptions of egoism, utilitarianism, libertarianism, and liberalism.

### Limits of This Study and Future Vision: Subject/Object Citizenship, Justice, and Common Good

The correlational analyses in the two surveys, thus, demonstrated the relative plausibility of the communitarian assumption. Moreover, as the constitution of the parent population in the two surveys is different about several factors such as residency, sex, age, marital status, these results seem robust. Moreover, it is demonstrated that these attributes do not influence the results.

Nevertheless, other factors, such as living countries, dates, political regimes, and culture, may affect the results, and similar comparative studies will be desirable in the future. Furthermore, these surveys were conducted in 2020 and 2021, historical years of the pandemic in the world. Therefore, studies during usual times will be desirable to remove the possible influence of such unusual global environments.

In addition, there can be a following methodological argument. The political philosophies are examined by people’s perceptions concerning citizenship and justice. On the other hand, normative philosophies such as libertarianism and liberalism are independent of people’s subjective beliefs. Thus, the results indicate only that communitarianism is most close to people’s popular belief so that the results do not increase its plausibility.

Nonetheless, the most critical methodological limit is perhaps that this survey measures citizenship and justice by the subjective recognition of respondents. This issue is well-known regarding subjective well-being, and there has been a long history of arguments and empirical verifications. In the same vein, replies concerning citizenship and justice signify the subjective recognition of the respondents that there is some level of citizenship or justice. The answers do not guarantee that these reflect objective situations. For example, if some replied that there is no disparity and distributive justice in Japan, this answer can be objectively wrong. The persons may be simply ignorant of the reality concerning the objective large inequality because of their low political consciousness, or media manipulation by governments may delude the persons in the worst cases. If many people believe that there are few problems regarding justice and citizenship in their countries by these factors, they may be happy at least during some periods. This situation may be a fool’s paradise.

The statistical analysis this study offers may conclude that justice and citizenship influence well-being in such a situation. However, this may be neither desirable nor sustainable. Therefore, it would be essential to discern whether the high degree of well-being is the consequence of blinding effects or objective increase in citizenship and justice. The best way to do this would be through the use of both objective and subjective indicators. While this study explored “subjective citizenship” and “subjective justice”, the development of measuring “objective citizenship” and “objective justice” is also valuable. There have been some related attempts such as social justice index (Bertelsmann Stiftung, 2019); Amartya Sen and Martha Nussbaum’s capability approach (and the Human Development Index of the United Nations Development Programme: section “Capability Approach: Consequential, Perfectionist, and Political Liberalism” in the previous paper) and John Rawls’ concept of primary goods may be able to function regarding justice (Rawls, 1971; Brighhouse and Robeyns, 2010).

In the same way, the common good in political philosophy signifies both the objective benefits of all concerned and the corresponding subjective benefits. The two can be distinctively expressed as the common goods and the common good. In other words, these are “objective common good” and “subjective common good”.

**FIGURE 1:**
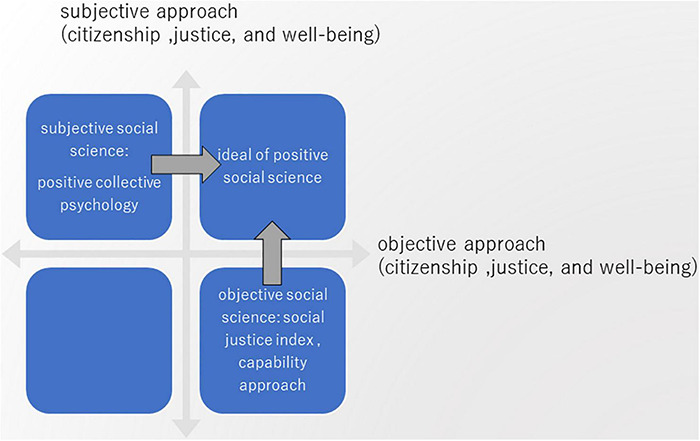
Subjective and objective social sciences.

Figure 1 indicates both as two axes, and the subjective approach of positive collective psychology in this study explores the subjective upper half; the objective approaches mentioned above correspond to the right half. These two can be called “subjective social science” and “objective social science,” respectively. Then, the concomitant use of both approaches is the ideal of positive social science. This is situated in the first quadrant, and the subjective and objective approaches can pursue it by reinforcing the other. These approaches based on this perspective will enable us to explore the vast frontier of positive social sciences.

### Prospect of Philosophical Psychology for Collective Well-Being and Common Good

In conclusion, this study demonstrated that citizenship, justice, and well-being (including political well-being) are substantially interdependent. This result coincides with the communitarian assumption; therefore, this assumption is most plausible among assumptions of main political philosophies.

As discussed in sections of the “Psychological Examination of the Plausibility of Political Philosophies Concerning their Assumptions” this reliability does not necessarily imply that communitarianism is the most credible political philosophy, because there can be other assumptions, analyses of which may lead to different plausibility concerning each political philosophy. Moreover, since other analyses, such as confirmatory factor analyses, can be conducted by constructing models on political philosophies, these may confirm or deny the abovementioned results and add new findings on this theme.

Nonetheless, the interdependence of the three concepts is essential for political philosophy, and this result can be considered to increase the plausibility of communitarian political philosophy as a whole.

Thus, collaboration between political philosophy and empirical psychology can generate scientific and philosophical progress. In this case, research on positive political psychology substantiated the relationship among citizenship, justice, and well-being, guided by political philosophy. Moreover, the notion of collective well-being, including political well-being, can be regarded as an essential element of subjective common good: the former can function as one of the empirical measurements of the latter from now on. Thus, positive political psychology can approach empirical inquiry for the common good.

On the one hand, this analysis empirically proved the plausibility of the communitarian assumption. Furthermore, it increases the reliability of communitarianism, because these three conceptions are closely associated with the core of the communitarian political philosophy. This outcome is a contribution of philosophical psychology, empirical psychology led by philosophy.

On the other hand, the empirical finding will enable political philosophy to advance if corroborated by scientific analyses. This vision indicates the possibility of psychological philosophy, political philosophy led by psychology.

As communitarianism regards the common good as the purpose of politics, the possible outcome arising from this research is the emergence of psychological, political philosophy for the common good. Moreover, since the common good implies increase in the collective well-being of people, positive political psychology can work as a psychology for the common good with the collaboration of political philosophy.

## Data Availability Statement

The datasets presented in this article are not readily available because the dataset of the first survey presented in this article is not readily available: the approval of the institute which conducted the survey is required. In contrast, the dataset of the second survey is available. Requests to access the datasets should be directed to MK, masaya_kobayashi@nifty.com.

## Ethics Statement

Ethical review and approval was not required for the study on human participants in accordance with the local legislation and institutional requirements. The patients/participants provided their written informed consent to participate in this study.

## Author Contributions

The author confirms being the sole contributor of this work and has approved it for publication.

## Conflict of Interest

The author declares that the research was conducted in the absence of any commercial or financial relationships that could be construed as a potential conflict of interest.

## Publisher’s Note

All claims expressed in this article are solely those of the authors and do not necessarily represent those of their affiliated organizations, or those of the publisher, the editors and the reviewers. Any product that may be evaluated in this article, or claim that may be made by its manufacturer, is not guaranteed or endorsed by the publisher.

## Footnotes

1 Representative literatures were enumerated in my “Political Philosophies and Positive Political Psychology: Inter-disciplinary Framework for the Common Good,” especially pp. 3–6. The particularly useful introductions of contemporary political philosophy and the debate between liberalism and communitarianism are, respectively, as follows: Sandel (2009) and Mulhall and Swift (1996).

2 The first survey was conducted by Mitsubishi Research Institute under my advice. The second survey was conducted with my research funds.

3 The question of economic well-being concerning the present in I COPPE is “When it comes to your economic situation (on which number do you stand now?).” Similarly, the questions of political well-being begin at “When it comes to your surrounding political situation.”

4 SAT: average of life satisfaction, satisfaction before coronavirus disease-2019 (COVID-19), and satisfaction within the problem of COVID-19 (first survey); life satisfaction and general satisfaction (or content) (second survey). HAP: average of overall happiness, overall happiness before COVID-19, and overall happiness within the problem of COVID-19 (first survey); overall happiness (second survey).

5 The hybrid character of the SWLS was reported by Huta (2016, pp. 222–223), and this finding was confirmed by our unpublished research.

6 The correlations between the SAT of the justice/citizenship are 0.475/0.431, and the former is slightly higher than the CV. This result is different from the first survey, although this is not so conspicuous that it does not change the general tendency mentioned here.

7 This variable is controlled only in the second survey because there is no data in the first survey.
