# Supplementary material for: Psychological Examination of Political Philosophies: Interrelationship Among Citizenship, Justice, and Well-Being in Japan
Source: Front Psychol. 2022 Feb 28;12:790671. doi: 10.3389/fpsyg.2021.790671 (PMC8919993; doi:10.3389/fpsyg.2021.790671)
Supplement: Supplementary file 1 [file Table_1.docx]

Appendix Table 1.


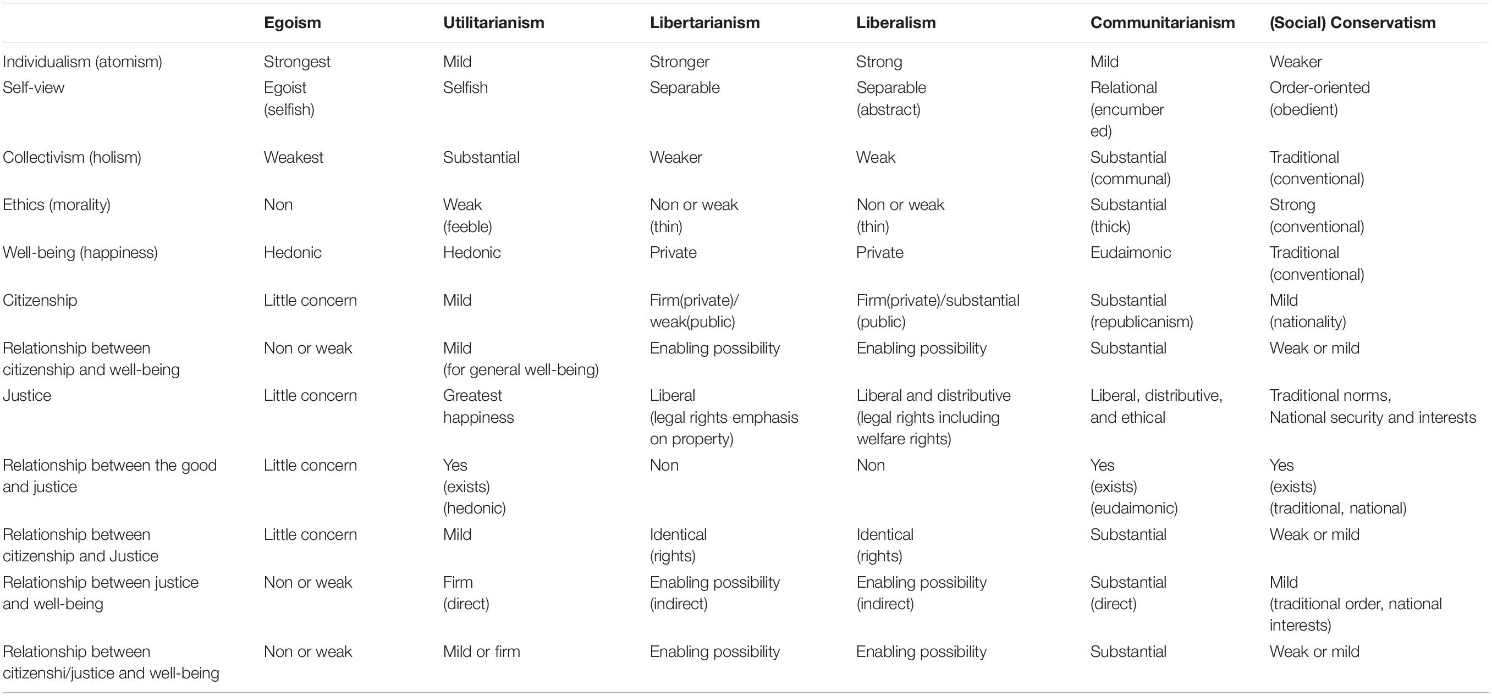


Note: Masaya Kobayashi, "Political Philosophies and Positive Political Psychology: Inter-Disciplinary Framework for the Common Good,"

Front. Psychol., 13 December 2021 | <https://doi.org/10.3389/fpsyg.2021.727818>, Table1.
